# Development of biodegradable films using sunflower protein isolates and bacterial nanocellulose as innovative food packaging materials for fresh fruit preservation

**DOI:** 10.1038/s41598-022-10913-6

**Published:** 2022-04-28

**Authors:** Maria-Nefeli Efthymiou, Erminta Tsouko, Aristeidis Papagiannopoulos, Ioanna-Georgia Athanasoulia, Maria Georgiadou, Stergios Pispas, Demetres Briassoulis, Theofania Tsironi, Apostolis Koutinas

**Affiliations:** 1 Department of Food Science and Human Nutrition, Agricultural University of Athens, Iera Odos 75, 11855 Athens, Greece; 2 Theoretical and Physical Chemistry Institute, National Hellenic Research Foundation, 48 Vassileos Constantinou Ave., 11635 Athens, Greece; 3 Department of Natural Resources and Agricultural Engineering, Agricultural University of Athens, 11855 Athens, Greece

**Keywords:** Biomaterials, Biomaterials, Environmental biotechnology, Nanobiotechnology

## Abstract

This study presents the valorization of side streams from the sunflower-based biodiesel industry for the production of bio-based and biodegradable food packaging following circular economy principles. Bacterial cellulose (BC) was produced via fermentation in 6 L static tray bioreactors using nutrient-rich supplements derived from the enzymatic hydrolysis of sunflower meal (SFM) combined with crude glycerol as carbon source. Novel biofilms were produced using either matrices of protein isolates extracted from sunflower meal (SFMPI) alone or SFMPI matrices reinforced with nanocellulose biofillers of commercial or bacterial origin. Acid hydrolysis was employed for ex-situ modification of BC to nanostructures (56 nm). The biofilms reinforced with bacterial nanocellulose structures (SFMPI-BNC) showed 64.5% higher tensile strength, 75.5% higher Young’s modulus, 131.5% higher elongation at break, 32.5% lower water solubility and 14.1% lower water vapor permeability than the biofilms produced only with SFMPI. The biofilms were evaluated on fresh strawberries packaging showing that the SFMPI-BNC-based films lead to effective preservation at 10 °C considering microbial growth and physicochemical profile (weight loss, chemical characterization, color, firmness and respiration activity). The SFMPI-BNC-based films could be applied in fresh fruit packaging applications.

## Introduction

The conventional biodiesel industry could be restructured into a novel biorefinery producing antioxidants and protein isolates from sunflower meal (SFM) and fermentation products, such as bacterial cellulose (BC), from crude glycerol^1,2^. The sustainable development of biorefineries will only be achieved through the production of marketable bio-based end-products that could compete with petroleum-derived counterparts. The production of biocomposite films for food packaging applications constitutes a market segment of high interest due to environmental and societal concerns on end-of-life management of conventional plastics, gradual depletion of fossil resources and sustainability issues^3,4^.

Proteins and plant or bacterial cellulose derivatives in the form of nanofibers, nanowhiskers or nanocrystals could be used in food packaging applications^5^. The conformational denaturation characterizing proteins is highly associated with their film-forming capacity, while the specific structural modification process employed could lead to targeted functional properties of the final protein-based formulation^6^. Protein isolates extracted from SFM (SFMPI) are non-toxic and possess an amino acid profile that meets Food and Agriculture Organization compliances and thus they could be utilized for edible films and coating formulations^7^. Salgado et al.^8^ employed the casting technique to produce sunflower protein concentrate (SFPC)-based films supplemented with essential oil and evaluated their preservation capacity on refrigerated sardine patties. The physico-chemical, mechanical and barrier properties of the biofilms showed slight variation with water solubility, the antioxidant capacity was enhanced and the elongation at break was decreased. The produced biofilms exhibited a positive effect on the auto-oxidation process of fish lipids and also contributed to the partial delay of growth of the total mesophilic microorganisms.

BC has superior physicochemical properties to plant-derived cellulose and it can be produced from crude renewable resources via fermentation^9^. The conversion of BC into bacterial nanocellulose structures (BNC) as biofillers in edible food packaging films could improve their physical and mechanical properties providing also desirable gas barrier properties, food quality and shelf-life^10,11^. Nanocellulose structures, including BNC, could improve oxygen and water vapor barrier properties^12^. Literature-cited publications have shown that biofilm production using nanocellulose and proteins exhibit better properties in terms of tensile strength, Young’s modulus, solubility and water vapor permeability than protein-based biofilms^13–16^, due to bonding synergies that occur between the reinforcing filler and the biopolymeric matrix. However, the applicability of such BNC-based biofilms in food packaging of fresh products is limited.

Glycerol could be used as plasticizer in biocomposite films production^17^. Crude glycerol derived from biodiesel production processes could be used as carbon source for BC production and as plasticizer in biocomposite films for food packaging applications. Approximately 66% of global glycerol production is derives from the biodiesel industry, generating 1 kg of glycerol for every 10 kg biodiesel produced^18–22^. Global biodiesel production is expected to reach 46 × 10^6^ m^3^ by 2025^23^, while conventional glycerol applications do not absorb surplus production.

This study focuses on the utilization of crude co-products (i.e. crude glycerol, SFMPI, BNC) derived from a novel sunflower-based biorefinery for the production of sustainable biodegradable films in order to substitute for conventional packaging materials following circular bio-economy principles. The properties of biofilms produced with crude streams have been compared with biofilms produced with commercial BNC. The biofilms have been evaluated as packaging films of fresh strawberries via quality and shelf life of the strawberries over storage. This study demonstrates that crude biorefinery-derived streams could be used for the production of biofilms that are suitable for packaging applications of fresh food products.

## Results and discussion

### Extraction of SFMPI and production of bacterial cellulose

The biorefinery concept presented by Efthymiou et al.^2^ has been employed for the extraction of SFMPI from SFM at a purity of 91.7% (w/w, db). SFM was also used for the production of crude enzymes (mainly protease) via solid state fermentation (SSF) of *Aspergillus awamori* that were subsequently employed in the hydrolysis of SFM solids derived after the extraction of protein isolate. The SFM hydrolysate was a nutrient-rich fermentation supplement with 1000 mg/L free amino nitrogen (FAN) concentration achieved after 72 h of enzymatic hydrolysis. The SFM hydrolysate (350 mg/L initial FAN concentration) was combined with decanted crude glycerol (20 g/L) as fermentation medium for BC production by *Komagataeibacter sucrofermentans* in static tray cultures. After 15 days, 12.0 ± 0.5 g/L BC were produced with 0.6 g BC/g glycerol conversion yield and 0.8 g/(L day) productivity. Around 68.3% of the initial FAN concentration was consumed. The achieved BC production efficiency ranks high among literature-cited studies using crude renewable resources^1,24^ indicating that it could lead to a cost-competitive process as compared to conventional bioprocesses where fermentation media account approximately for 30% of the total production cost^25^.

### Bacterial nanocellulose production

The produced BC was ex-situ modified via 50% H_2_SO_4_ treatment for 48 h at 55 °C. The *ζ*-potential of the obtained BNC was − 34.1 ± 0.3 mV, similar to the values reported by Yan et al.^26^ (− 34.8 mV) and Rollini et al.^27^ (− 33.0 mV) employing also H_2_SO_4_. Vasconcelos et al.^28^ reported *ζ*-potential values ranging from − 24.7 to − 53.6 mV depending on the acid concentration, treatment duration and acids (H_2_SO_4_/HCl) used. The *ζ*-potential represents the surface charge density of the final formulation, while absolute values higher than 30 mV generally indicate good stability of BNC water dispersions. HCl-assisted hydrolysis of cellulose generates practically neutral nanocrystals, while H_2_SO_4_ hydrolysis generates strongly charged nanoparticles due to the fact that glucose units are functionalized with sulfate ester groups^29^. Nanocrystalline cellulose (NCC) showed much lower *ζ*-potential value (− 15.1 mV) compared to BNC. Higher absolute values (− 25.9 to − 20.6 mV) have been reported for NCC derived after acid-assisted treatment of roselle-derived microcrystalline cellulose (MCC) under various hydrolysis time^30^.

The dynamic light scattering (DLS) of BNC showed two peaks confirming the BNC polydispersity (Fig. S1). The peak centred at 56 nm corresponds to BNC nanoparticles. The second peak at 385 nm corresponds to aggregates of BNC nanoparticles that are possibly induced by intermolecular hydrogen bonding^27^. BC nanoparticles of 240 nm with BNC aggregates of 1030 nm have been reported when BC was subjected to 65% (w/w) H_2_SO_4_ treatment at 55 °C for 2 h^27^. The size distributions extracted by CONTIN are weighted by the scattered intensity of the different species in solution, which depends strongly on size and thus the number-weighted size distribution is expected to be mainly represented by small-size particles. In the case of NCC, the R_h_ (44.6 nm) was found slightly lower compared to BNC (56 nm) (Fig. S1). Cui et al.^31^ demonstrated that the length of NCC prepared via ultrasound-assisted enzymatic hydrolysis from wheat MCC was shifted towards lower values with prolonged enzymatic hydrolysis and ultrasonication time. NCC subjected to 120 h of enzymatic hydrolysis and 60 min of ultrasonication showed length values of 50–80 nm. Kian et al.^30^ investigated the isolation of NCC from roselle-derived MCC via H_2_SO_4_ hydrolysis. The average length size of NCC varied between 553–281 nm with acidic pretreatment from 30 to 60 min.

The X-ray diffraction (XRD) pattern of BNC exhibited three diffraction peaks at 2θ angle of 14.8°, 16.9° and 22.7°, which normally correspond to (1 0 0), (0 1 0) and (1 1 0) cellulose crystalline domains of the I_α_ allomorph or (1 0), (1 1 0), and (2 0 0) planes of type I_β_ (Fig. S2). *Acetobacter xylinus* produces BC consisting mainly of the I_α_ allomorph^32^. The crystallinity index of BNC was 88.3% (Fig. S2), while slightly higher crystallinity indices (89.6–91.0%) have been obtained for cellulose nanocrystals derived from acid hydrolysis of BC produced by acetic acid bacteria^26,28,33^.

### Fourier transform infrared spectroscopy (FTIR) analysis

**Figure 1:**
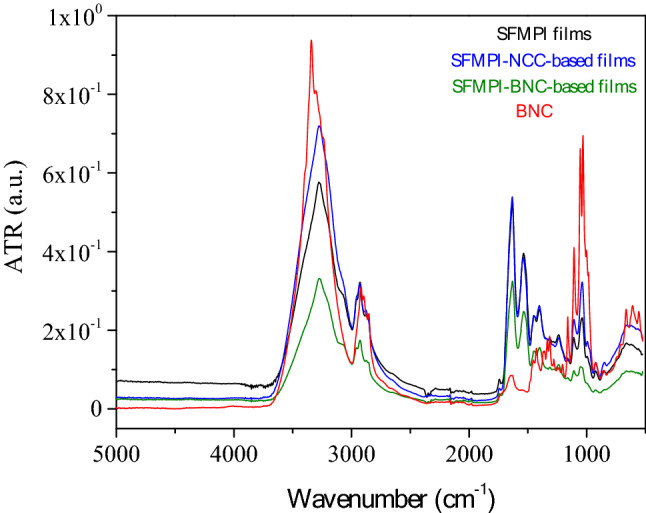
FTIR spectra of BNC derived after the acid hydrolysis of bacteria cellulose, SFMPI biofilms, SFMPI-NCC- and SFMPI-BNC-based biofilms.

All samples presented an absorbance band at 3000–3700 cm^−1^ (Fig. 1 and Table S1) that corresponds to stretching vibration of OH groups and further reflects the water-water and water-biopolymer interactions^34^. Furthermore, N–H stretching vibration of hydrogen bonded amides (amide A) have been reported to absorb within the same region^35^. The peaks that appear within the band 2700–2995 cm^−1^ are attributed to C–H stretching of the cellulose backbone^34^ and C–H stretching from the protein samples^36^.

Considering the FTIR spectra of BNC, there was a peak of strong intensity at 1159 cm^−1^ attributed to asymmetric stretching vibrations from C–O–C^28,37^. Martelli-Tossi et al.^14^ reported that peaks in the region 1155–1099 cm^−1^ were only found in the spectrum of cellulose nanocrystals (CNCs) but not when cellulose nanofibers (CNFs) were considered. This consideration in combination with the DLS analysis performed in this study (Fig. S1) indicates that BC hydrolysis resulted in cellulose nanostructures in the form of nanocrystals. Peaks at 1232 cm^−1^, 1243 cm^−1^ and 1276 cm^−1^ correspond to out of plane bending vibration of C–O–H at C6 and at 1203 cm^−1^ reflecting symmetrical stretching vibration from C–O–C^37^. The peak at 1052 cm^−1^ was detected only in the FTIR spectra of BNC corresponding to vibrations of asymmetrical C–H and stretching of C–O related to the β-glycosidic linkages between β-d-glucopyranoses in cellulose^14,28^.

The FTIR spectra of biofilms showed three characteristic peaks at 1240 cm^−1^ (C–N stretching of amides), 1536 cm^−1^ (N–H bending of amides) and 1633 cm^−1^ (C=O stretching of amide) that correspond to amide bands (Table S1). These peaks can be further associated with the presence of protein isolates in the biopolymeric matrices^35,38^.

### Physical characterization of biofilms

#### Thickness evaluation

The thickness of SFMPI-based (229.6 μm) and SFMPI-NCC-based (227.9 μm) films (Table 1) did not show significant differences (*p* < 0.05), while significant lower value (187.4 μm) was obtained for SFMPI-BNC-based films. These differences may be attributed to the way that protein isolates and cellulose nanoparticles interact within the biopolymer matrix^43^.

**Table 1:** Mechanical and physical properties of SFMPI-, SFMPI-NCC- and SFMPI-BNC-based biofilms compared to the properties of renewable cellulose or commercial cellulose derivatives based films, cited in the literature.

Biofilms	Tensile strength (MPa)	Elongation at break (%)	Young’s modulus (MPa)	Thickness (μm)	Water vapor permeability (g/m·s·Pa)	Solubility (%)	Source
SFMPI	1.8 ± 0.4^a^	33.3 ± 8.4^a^	42.6 ± 8.9^a^	229.6 ± 11.4^a^	2.9 × 10^–10^ ± 1.5 × 10^–11a^	52.8 ± 0.43^a^	This study
SFMPI-NCC	1.7 ± 0.2^a^	64.9 ± 10.9^b^	46.4 ± 4.8^b^	227.9 ± 9.4^a^	2.0 × 10^–10^ ± 1.5 × 10^–11b^	27.3 ± 0.55^b^	
SFMPI-BNC	3.0 ± 0.4^b^	77.0 ± 7.5^c^	74.8 ± 17.8^c^	187.4 ± 8.9^b^	2.5 × 10^–10^ ± 0.0^c^	32.5 ± 0.18^c^	
Gelatin	~ 16.0	~ 17.0	~ 440.0	65 ± 13	–	–	^13^
Gelatin/CNC from eucalyptus	~ 12.0–17.0	~ 6–14	~ 430.0–500.0	65 ± 13	–	–	
Soy protein isolate (SPI)	6.1	18.0	4.6	62 ± 11	12.9 × 10^–10^	26	^14^
SPI/nanocellulose from licorice	11.2	63.8	–	–	1.4–1.9 × 10^–10^	–	^38^
SPI/soybean straw CNF	9.0	8.0	5.7	80	7.0 × 10^–10^	33	
SPI/ soybean straw CNC	8.4	4.2	5.4	82	14.0 × 10^–10^	20	
SPI	3.4	132	–	365	–	–	^39^
SPI/MCC	5.2	68.0	–	296	–	–	
Whey protein	~ 2.2	~ 24.0	~ 69.0	119	0.2 × 10^–10^	37.5	^15^
Whey protein/nanocellulose from oat husks	~ 4.3	~ 11.0	~ 100.0	136	0.1 × 10^–10^	39.2	
SPI	1.1	66.0	26.9	–	4.3 × 10^–10^	37.8	^16^
SPI/ starch nanocrystals	1.34	58.6	39.4	–	4.8 × 10^–10^	21.6	
SPI	~ 2.6	~ 95	45.3	193	–	60.1	^40^
Gelatin	~ 1.8	~ 95	66.1	277	–	33.4	
SPI + Gelatin/MCC	5.9	25.7	120.0	293	-	28.1	
Gelatin/BNC	83.7–108.6	33.7–23.2	2189.5–2350.4	–	–	–	^41^
SFPI	4.0	24.0	0.58	74–80	2.0 × 10^–10^	–	^42^

Different superscript letters within same column indicate statistically significant differences (*p* < 0.05).

#### Solubility evaluation

Significant solubility reduction was observed in SFMPI-NCC-based (27.3%) and SFMPI-BNC-based (32.5%) films as compared to SFMPI films (52.8%). NCC has lower particle size than BNC increasing the contact surface area per volume with the SFMPI matrix and thus more hydrogen bonding interactions occur contributing to decreased solubility in water. Therefore, in the case of NCC-based films the availability of hydroxyl groups that interact with H_2_O molecules is lower leading to reduced solubility capacity of the produced films. The effect of particle size of reinforcing agents on interactions between OH-H_2_O that further reflect the solubility capacity of the final material, has been previously confirmed by Martelli-Tossi et al.^14^. Specifically, the authors reported that SPI-based biofilms reinforced with CNFs of higher size derived from soybean straw, showed higher solubility (33%) than films containing nanosized particles of CNCs (20%) obtained after enzymatic hydrolysis of soybean straw. Solubility values in literature-cited biofilm formulations reinforced with cellulose nanoparticles vary between 20 and 40% (Table 1).

#### Water vapor permeability

The water vapor permeability (WVP) of the three biofilms (2.0–2.9 × 10^–10^ g/m·s·Pa) showed statistically significant differences (*p* < 0.05). SFMPI-NCC- and SFMPI-BNC-based films showed 31.0% and 13.8% lower WVP than SFMPI-based films. The incorporation of cellulose nanodomains into the SFMPI matrix led to improved water barrier properties due to better molecular arrangement, the crystalline nature of nanocellulose and probably reduced sorption and diffusion coefficients of the SFMPI^34,41^. The formation of a denser matrix by the incorporation of biofillers might have eliminated water vapor transmission phenomena leading to low WVP. The lower WVP of NCC-based films than BNC-based films could be attributed to the lower particle size of NCC leading to better dispersion capacity. The NCC crystallinity index (89.0%) is slightly higher than BNC (88.3%), which may have limited further the water vapor diffusion in SFMPI-NCC-based biofilms^34,41^.

The water vapor transfer rate (WVTR) of SFMPI- (2.69 × 10^–3^ g/s·m^2^), SFMPI-NCC- (1.84 × 10^–3^ g/s·m^2^) and BNC-based (2.84 × 10^–3^ g/s·m^2^) films produced in this study were lower than WVTR values of polyvinylidene chloride (0.02 × 10^–3^ g/s·m^2^), polyethylene (0.12 × 10^–3^ g/s·m^2^) and plasticized polyvinyl chloride (0.08 × 10^–3^ g/s·m^2^) based films (thickness in the range of 12.7–18.3 μm)^12^. Thus, the WVTR of the biofilms produced in this study are higher than commercially available films derived from fossil resources. This could be advantageous in the case of packaging of fresh food products (up to specific thresholds) where optimal water vapor removal from the packaging headspace could lead to lower microbial spoilage and quality deterioration of the packed food.

### Mechanical characterization of biofilms

The stress strain curves demonstrated that elongation at break and Young’s modulus (Table 1) in SFMPI-NCC- and SFMPI-BNC-based films were higher than SFMPI-based films at statistically significant levels (*p* < 0.05). The Young’s modulus of SFMPI-NCC- and SFMPI-BNC-based films were 9.0% and 75.5% higher than SFMPI-based films, while the respective elongation at break values were 94.9% and 131.5% higher than SFMPI-based films. García-Ramón et al.^34^ also reported that the elongation at break of neat banana starch films (7.7%) was lower that the respective values (up to 19.9%) achieved when banana starch films were reinforced with different concentrations of plant cellulose nanoparticles (1.75–4%). In some literature-cited studies, the reinforcement of biofilm matrices with cellulose nanostructures led to lower elongation at break due to the heterogeneous matrix of the biocomposite, which partly impedes interfacial stress transfer resulting in self-harden^14–16,39^. Kang et al.^39^ reported that elongation at break of SPI-based films gradually decreased from 132 to 68% when the films were reinforced with increasing MCC concentration up to 0.1% (w/w). The tensile strength of SFMPI-based and SFMPI-NCC-based films did not show any statistically significant difference, while BNC incorporation significantly enhanced tensile strength with an increase of 64.6% as compared to SFMPI-based films.

The SFMPI-BNC-based films showed higher tensile strength (3 MPa), Young’s modulus (74.8 MPa) and elongation at break (77.0%) demonstrating a flexible and consolidated network that could be attributed to hydrogen bonding interactions of filler-filler and filler-matrix between BNC and the protein molecules. The nano-sized network of BNC is highly efficient in stress transfer phenomena that occur from the polymeric chains to the nanoparticles of BNC leading eventually to improved overall mechanical behaviour of the nanocomposite films. Τhe hydrophilic nature of both BNC and SFΜPI facilitates the miscibility between these two components contributing to the production of biofilms with improved performance^34^. George and Siddaramaiah^41^ reported the production of gelatin nanocomposite films supplemented with 1–5% (w/w) BNC produced by *Gluconacetobacter xylinus* with higher tensile strength (83.7–108.6 MPa) and Young's modulus (2189.5–2350.4 MPa) than the respective values achieved in this study with SFMPI-BNC-based biofilms (Table 1). However, the elongation at break reported for gelatin-BNC-based biofilms by George and Siddaramaiah^41^ was 2.3–3.3 fold lower than the SFMPI-BNC-based films produced in this study (Table 1).

This study employed SFMPI of 91.7% purity as biofilm matrix. The protein source and purity affect the biofilm properties. For instance, the impurities that remain after the extraction of SFMPI (e.g. phenolic compounds) may lead to weak tensile strength and Young’s modulus values when compared to commercial sources of proteins^42^. High impurity levels (> 10%) of protein concentrate preparations may interact with the filler and thus hinder modulus and flexibility properties of the final biofilm formulations. Acquah et al.^36^ reported that the biofilms produced with pea protein isolates resulted in 3.6 fold higher tensile strength (0.6 MPa) and 6.8 fold higher elongation at break (65.6%) compared to biofilms produced with pea protein concentrates. Young’s modulus was increased from 0.03 to 6.7 MPa when pea protein concentrates were substituted with isolates.

### Biofilm evaluation in fresh strawberry packaging

Evaluation of the biofilms as food packaging materials was conducted in freshly harvested strawberries during isothermal storage at 10 °C over a 15-day period. Conventional polyvinyl chloride (PVC) membranes were applied for comparison purposes. PVC films have been traditionally used in agriculture, as they reduce moisture loss by evaporation and increase the efficiency of water use^44^. So far, they have been proposed as appropriate packaging materials for the shelf life extension of strawberries, including preservation of color, and nutritional value (vitamin C), reduction of respiration rate and weight loss. More specifically, to reduce weight loss and further maintain fruit quality during storage, strawberries are routinely harvested by hand in polystyrene baskets which are manually wrapped with PVC films in the packing houses. Thus, issues related to safer products, sustainability, renewability and biodegradability have shifted global demand towards bio-based polymers discouraging conventional polymers i.e. PVC^45^.

This study aimed to introduce alternative packaging materials (films) appropriate for fruit preservation, which provide adequate mechanical barriers while enabling the development of a preservative headspace composition extending shelf life.

#### Weight loss

**Figure 2:**
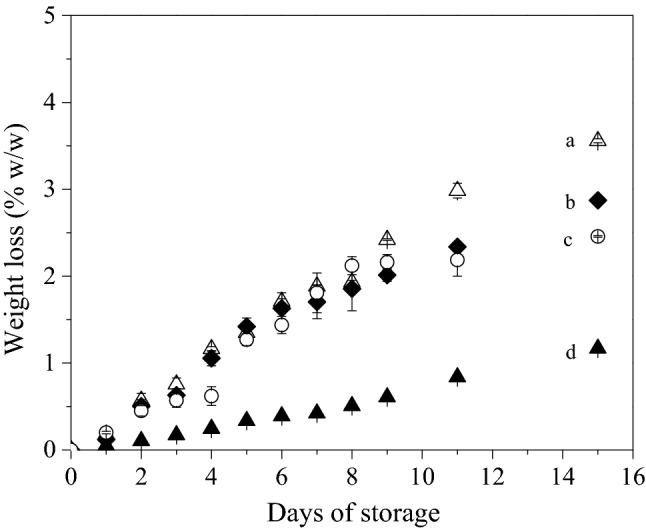
Weight loss (% w/w) of strawberries sealed with conventional PVC (▲), SFMPI (Δ), SFMPI-NCC (♦) and SFMPI-BNC (○) based biofilms over 15 days isothermal storage at 10 °C. Different letters indicate statistically significant differences (*p* < 0.05).

Strawberries show high susceptibility to water loss during storage, due to respiration and transpiration phenomena. Weight loss is associated to unattractive appearance of strawberry fruits. All strawberry samples exhibited weight loss within the acceptable limit of 6% (w/w), that has been set for maximum weight loss of fresh fruits to be considered as marketable products^46,47^. Weight loss gradually increased in all samples with maximum values observed at the end of storage (Fig. 2).

Water loss occurred from the fruit tissue due to high respiration rates and CO_2_ transfer within the fruit skin, resulting gradually to shrinkage and overall quality decay^48^. Weight losses at the end of storage (15th day) was significantly affected depending on the biofilm employed, while Tukey test revealed significant differences at every level of comparison. The weight losses recorded in this study were similar to the respective values reported by Duarte-Molina et al.^49^ and Khodaei et al.^50^ for fresh strawberries stored under refrigeration (4–6 °C) for 8–16 days. The variations in the weight reduction of strawberries sealed with conventional PVC and the tested alternative biofilms was mainly attributed to the differences in the water vapor permeability as described in section “Water vapor permeability”.

#### Respiration activity

**Figure 3:**
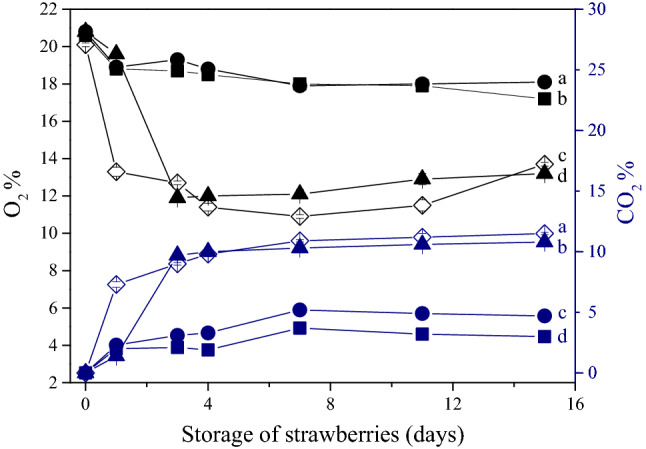
Respiration activity in terms of O_2_ (black) and CO_2_ (blue) during 15 days of isothermal storage of strawberries at 10 °C in containers sealed with conventional PVC (◊), SFMPI (▲), SFMPI-NCC (●) and SFMPI-BNC (■)-based films. Different letters for the same evaluation factor indicate statistically significant differences (*p* < 0.05).

The alterations in O_2_ and CO_2_ concentration in the headspace of containers sealed with conventional PVC, SFMPI, SFMPI-NCC and SFMPI-BNC-based biofilms storage are illustrated in Fig. 3.

In all cases, O_2_ concentration decreased from the early stages of storage, while CO_2_ concentration gradually increased in the headspace of all the applied films until the 7th day, without significant differences thereafter (*p* < 0.05). At the end of storage, final levels of O_2_ and CO_2_ obtained for all films showed statistically significant differences with all the possible combined pairs being different. Higher CO_2_ levels may result in inhibition of the formation of aerobic metabolites and transition from aerobic to anaerobic metabolism (< 3% O_2_ levels). This is the main factor that leads to the development of undesired off-flavor, which is a common issue in the case of passive or modified atmosphere packaging of fresh fruits^51^. In the present study, relatively high CO_2_ levels were recorded in the case of SFMPI films (with the advantage of the bacteriostatic and fungistatic activity of CO_2_ resulting in shelf life extension), while maintaining high O_2_ concentrations in the package headspace prevents the development of anaerobic conditions. The equilibrium between O_2_ and CO_2_ depends on films’ gas permeability, storage temperature, respiration rate of the packed fruits, surface areas of films and fruits and finally headspace volume to product mass ratio^52^.

#### Chemical evaluation of strawberry quality

**Figure 4:**
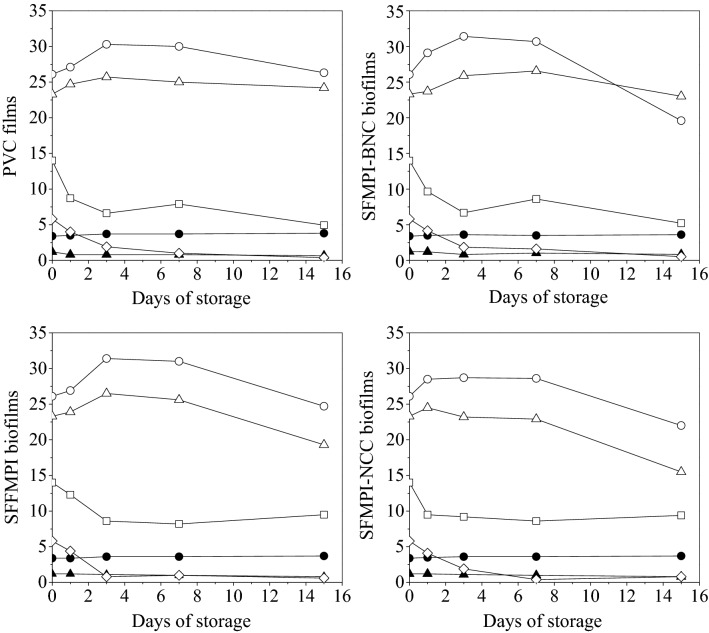
Time course of pH (●), TTA (▲), glucose (∆), fructose (○), sucrose (◊) and citric acid (□) during storage of strawberries in containers sealed with conventional PVC, SFMPI, SFMPI-NCC and SFMPI-BNC-based biofilms over 15 days at 10 °C.

Individual sugar levels along with pH, total titratable acidity (TTA) and citric acid content are shown in Fig. 4. The pH of the samples increased slightly during storage while TTA (expressed as citric acid equivalents) decreased. The fresh strawberries used in this study presented a humidity of 88.2 ± 0.9% w/w (wet basis, wb) and 55.2% g/g (dry basis, db), sugars including sucrose (5.8% w/w, db), glucose (23.3% w/w, db) and fructose (26.1% w/w, db). These data are in accordance with Giampieri et al.^53^ who reported respective values of 91% w/w (wet basis) and 54.0% w/w (db). The initial citric acid content was 14.0% w/w (db) (Fig. 4). During the tested period, strawberries sealed with conventional PVC membrane, neat SFMPI-, and SFMPI-BNC-based biofilms showed an increase in the total sugars content up to the 3rd day of storage reaching 57.9% (w/w, db), 58.8% (w/w, db) and 59.1% (w/w, db) respectively while total sugars decreased thereafter. In the case of strawberries sealed with SFMPI-NCC-based biofilm, total sugars increased after 1 day of storage (57.1% w/w, db) while they were gradually reduced at the end of storage. The major sugar in all ripening stages of strawberries has been reported to be glucose^54^ while in the current study fructose was determined as the predominant one. Sucrose content decreased throughout storage, reaching values of 0.4%, 0.6%, 0.8% and 0.5% (w/w db) for PVC, SFMPI-, SFMPI-NCC- and SFMPI-BNC-sealed samples respectively. Lower levels of sucrose during storage are associated with the activity of invertase, which is higher in ripe fruits compared to green fruits, leading to sucrose reduction and simultaneous increase of glucose and fructose^54^. Glucose increased during the early stages of storage (1–3 days) in all the examined cases while glucose content was reduced thereafter (Fig. 4). A similar trend was observed for fructose with its content being increased or remaining almost stable until the 7th day of storage. Based on the analysis of organic acids, citric acid was the sole detectable acid. Citric acid content is directly related to the acidity of fruits^55^. The citric acid content sharply decreased during the early stages of storage, remaining almost stable thereafter. The TTA was slightly decreased during storage (1.24–0.66%, w/w, wb). The pH values fluctuated between 3.4–3.8 in all the examined cases.

#### Color of packaged strawberries

Color evaluation (Table 2) was based on the analysis of factors including brightness (L), a*, b*, color saturation (C) and Hue angle (Hue). L-value showed a decreasing trend throughout storage for each applied biofilm (apart from PVC), without statistically significant differences (*p* < 0.05). This is associated with fruit darkening phenomena that occur during storage. Khodaei et al.^50^ associated the reduction of lightness of strawberries (refrigerated) with the mold contamination on the fruit surface during storage. The L-value was reduced by 10.5% (with respect to the initial values at day zero) in the case of PVC, while the corresponding reduction of L-values in the case of SFMPI-, SFMPI-NCC and SFMPI-BNC-based films was 1.7% and 3.0%, and 3.6% respectively, on the 15th day of storage. These alterations were found insignificant based on Tukey test. Reduced C-values in all cases indicated less vivid coloration of strawberries during storage. C-values reduced by 46.4%, 28.7%, and 19.2% for strawberries sealed with PVC, SFMPI- and SFMPI-NCC-based films respectively. Strawberries sealed with SFMPI-BNC-based films presented a more vivid coloration compared to the aforementioned (reduction of 8.6% with respect to the initial value at day zero). Overall external color changes in fully ripe strawberries were milder when SFMPI-, SFMPI-NCC- and SFMPI-BNC-based films were applied as compared to PVC.

**Table 2:** Color profile and firmness of strawberries in containers sealed with PVC, SFMPI, SFMPI-NCC and SFMPI-BNC films over 15 days storage at 10 °C.

Color							Firmness
Films	Day	L	a*	b*	C	Hue	F_max_ (N)
PVC	0	32.4 ± 1.1 a	25.3 ± 0.9 a	15.7 ± 0.3 a	30.0 ± 0.3 a	30.8 ± 0.9 a	3.2 ± 0.7
	7	28.9 ± 1.2 b	22.5 ± 1.0 b	9.9 ± 0.4 b	24.7 ± 1.2 b	22.9 ± 1.1 b	2.5 ± 0.5^a^
	15	29.0 ± 0.9 b^a^	22.1 ± 0.2 b^a^	11.6 ± 0.2 c^a^	16.1 ± 0.7 c^a^	27.6 ± 0.5 c^a^	1.6 ± 0.6^b^
SFMPI	0	32.4 ± 1.1 a	25.3 ± 0.9 a	15.7 ± 0.3 a	30.0 ± 0.3 a	30.8 ± 0.9 a	3.2 ± 0.7
	7	32.5 ± 1.5 a	24.7 ± 1.2 b	16.1 ± 0.6 b	29.8 ± 1.4 a	33.2 ± 1.4 b	2.5 ± 0.7^a^
	15	31.9 ± 0.8 a^a^	19.1 ± 0.8 c^b^	9.4 ± 0.5 c^b^	21.4 ± 0.9 b^b^	25.2 ± 0.5 c^b^	1.9 ± 0.5^c^
SFMPI-NCC	0	32.4 ± 1.1 a	25.3 ± 0.9 a	15.7 ± 0.3 a	30.0 ± 0.3 a	30.8 ± 0.9 a	3.2 ± 0.7
	7	31.6 ± 1.4 a	23.3 ± 1.2 b	12.9 ± 0.4 b	26.7 ± 1.2 b	28.2 ± 1.3 b	2.0 ± 0.4^a^
	15	31.5 ± 0.8 a^a^	21.0 ± 0.3 c^a^	11.8 ± 0.6 c^a^	24.2 ± 0.7 c^c^	29.0 ± 0.9 b^a^	1.7 ± 0.3^b^
SFMPI-BNC	0	32.4 ± 1.1 a	25.3 ± 0.9 a	15.7 ± 0.3 a	30.0 ± 0.3 a	30.8 ± 0.9 a	3.2 ± 0.7
	7	32.1 ± 1.6 a	23.9 ± 1.2 b	15.4 ± 0.6 b	28.5 ± 1.4 b	32.8 ± 1.1 b	2.2 ± 0.5^a^
	15	31.3 ± 1.3 a^a^	22.8 ± 0.8 c^a^	14.7 ± 0.3 c^c^	27.4 ± 0.7 b^d^	32.4 ± 0.6 b^c^	2.0 ± 0.1^c^

Color Evaluation: Different letters within same columns, same factor and same film applied indicate statistically significant differences. Different superscript letters amongst same factors, regarding the 15th day of storage applied indicate statistically significant differences (*p* < 0.05).

Firmness Evaluation: Different letters within same column (firmness for each applied film regarding the 7th and 15th day of storage) or at 15th day of storage (all films) indicate statistically significant differences (*p* < 0.05).

#### Firmness of strawberries

Firmness is an important indicator of strawberry quality and it is highly dependent on the fruit cultivar and stage of ripening. The firmness of the examined strawberries (Table 2) was 3.2 ± 0.7 N at time zero. Hwang et al.^55^ reported slightly higher firmness values (4.8 N) for ripe strawberries of the *Maehyang* cultivar. The firmness value of strawberries gradually decreased during storage. Duarte-Molina et al.^49^ reported reduction in strawberry firmness during storage at 6 °C for 8 days. According to Paniagua et al.^56,57^, fruit softening may be attributed to senescence, cellular breakdown and pectin hydrolysis or depolymerization. Comparing firmness values of strawberries sealed with all the applied films, between the 7th and 15th day of storage, statistically significant differences were detected (*p* < 0.05). Strawberries sealed with SFMPI-BNC-based films showed the highest firmness value (2.0 ± 0.1 N) at the end of storage. Tukey test revealed significant differences between SFMPI-BNC-based films and PVC or SFMPI-NCC-based ones.

#### Microbiological evaluation

The microbial load of total viable count and yeasts and molds in fresh strawberries during storage are depicted in Table 3 and Fig. S3a,b respectively. The obtained experimental data were fitted to the Baranyi growth model and the respective kinetic parameters at each tested packaging system were evaluated (Table 3). The initial load of total viable count and yeasts and molds ranged within 2.8–3.1 log CFU/g and 2.9–3.3 log CFU/g, respectively. These values are in agreement with the initial microbial load observed by Shahi et al.^58^ in fresh strawberries. Strawberries stored using the conventional PVC films exhibited higher microbial growth rates, compared to all biofilms. The estimated k values for the biofilms indicate very slow microbial growth in the packed strawberry samples, which may be attributed to their higher WVP as reported in section “Water vapor permeability” and thus, preventing high moisture concentrations in the package headspace which accelerate microbial (including yeasts and mold) activity.

**Table 3:** Growth rates (k in 1/days) and final population (N_max_ in logCFU/g) of total viable count and yeasts and molds in strawberries stored at 10 °C using conventional PVC and SFMPI-, SFMPI-NCC and SFMPI-BNC-based biofilms (Mean values ± standard error based on the statistical variation in the kinetic parameters of the Baranyi growth model-regression analysis).

	Total viable count		Yeasts and molds	
	k (1/days)	N_max_ (logCFU/g)	k (1/days)	N_max_ (logCFU/g)
PVC	1.444 ± 0.374	5.1 ± 0.1	1.825 ± 0.431	5.3 ± 0.2
SFMPI	0.147 ± 0.022	4.5 ± 0.1	0.336 ± 0.138	4.1 ± 0.2
SFMPI-NCC	0.248 ± 0.037	4.5 ± 0.1	0.330 ± 0.056	4.4 ± 0.1
SFMPI-BNC	0.275 ± 0.179	3.6 ± 0.2	0.094 ± 0.037	3.6 ± 0.1

#### Decay rate of packed strawberries

The strawberry samples were visually evaluated for decay indications, including discoloration, tissue softening or evident microbial growth. Brown spots or softened wound areas onto the fruit surface were also recorded. The decay rate of strawberry samples sealed with all the applied films is presented in Fig. S4.

All samples showed increasing decay rates after the 3rd day of storage. Samples sealed with PVC films showed the highest decay rate that reached up to 100% at the end of storage. In the case of SFMPI- and SFMPI-NCC-based biofilms, stronger preservative effect (50%) was recorded after 1 week of storage remaining stable thereafter. Strawberries sealed with SFMPI-BNC-based biofilms showed a decay rate of 60% after 7-day storage, while an increase of 14.3% was observed at the end of storage. According to Khodaei et al.^50^, the decay percentage of fresh strawberries reached values up to 59.6% after 16 days of storage at 4 °C.

## Conclusions

This study proposed the utilization of crude bio-based co-products (i.e. crude glycerol, BNC, SFMPI) derived from a sunflower-based biorefinery for the development of biodegradable food packaging. SFMPI was extracted from SFM, while BC was produced via *K. sucrofermentans* cultures using crude glycerol and SFM hydrolysates as fermentation media components. Ex-situ modification of BC led to BNC preparations used as reinforcing biofillers in SFMPI matrices. The moderate mechanical response and the inferior solubility, as well as water vapor barrier properties of the SFMPI-based biofilms compared to conventional polymers were enhanced by the incorporation of cellulose nanostructures in the protein matrix. The applicability of the produced biofilms as packaging materials was evaluated by monitoring various quality parameters of fresh strawberries in containers sealed with the produced biofilms during a long-term storage. Levels of O_2_ and CO_2_ of SFMPI-BNC-based biofilms demonstrated the establishment of a passive modified atmosphere inside the packages. The proposed biofilms could sustain packaging intended for fresh products offering deterioration of microbial spoilage and high quality.

## Materials and methods

### Raw materials

SFM and crude glycerol were provided by the biodiesel industry P.N. Pettas S.A. (Greece). Crude glycerol was decanted until a glycerol purity of 92.4% (w/w). The commercial NCC (NAVITAS o.d.d., Slovenia, EU) derived from tree cellulose was used as a reinforcing agent. The CrI was equal to 89.0% while width and length were 35 nm and 350 nm length respectively (defined by the company).

### Microorganisms

The production of crude enzymes was achieved via SSF using the fungal strain *Aspergillus awamori* 361U2/1. Maintenance, storage, sporulation and inoculation of the fungal strain have been described by Efthymiou et al.^2^.

BC was produced using the bacterial stain *Komagataeibacter sucrofermentans* DSM 15973 (Leinbniz-DSMZ Institute of Germany). Stock cultures and inoculum were prepared according to Tsouko et al.^1^.

### SFMPI production

SFM was initially subjected to ultrasound assisted extraction of phenolic compounds using 50% (v/v) aqueous ethanol at a solid to liquid ratio of 1:10 (w/v) for 30 min. The remaining solids were treated with 5 M NaOH until pH 10.5. The SFM suspension was subsequently acidified with 5 M HCl until the isoelectric point (pH 4.3). The methodology is described by Efthymiou et al.^2^. The SFMPI purity was determined via Total Kjeldahl Nitrogen analysis (Foss, Denmark)^2^.

### SFM hydrolysate production

SFM hydrolysis was carried out using crude enzymes produced via SSF conducted at 55% initial moisture, 30 °C and 95 h incubation using untreated SFM inoculated with a fungal spore suspension (1 × 10^7^ spores/g SFM). Aqueous extracts of SSF were sequentially used in hydrolysis of SFM that remained after the extraction of phenolic compounds and proteins^2^.

### BC production

BC production was conducted in static tray bioreactors (60 × 18 × 40 cm) at 30 °C for 15 days using 10% (w/w) inoculum with 1.5 L working volume. The fermentation medium contained 20 g/L decanted crude glycerol and SFM hydrolysate with 350 mg/L initial free amino nitrogen (FAN) concentration. BC cultures and its purification are described by Tsouko et al. ^1^.

### Bacterial nanocellulose production

The ΒC was lyophilized and comminuted using a hammer mill (Casella London). Acid hydrolysis of BC (10 g/L) was carried out using 50% (w/w) H_2_SO_4_ for 48 h at 55 °C and 500 rpm. Deionized water and 30% H_2_O_2_ were subsequently added at specific ratios^26^ under stirring until bleaching was completed. BNC suspensions were centrifuged (9000 rpm, 4 °C, 15 min), rinsed with deionized water and ultrasonicated (60 kHz, 300 W, Sonoplus 3200, Germany) for 3 min to remove the acid excess. The procedure was repeated two times^26^. BNC suspensions were neutralized using dialysis membranes (Medicell Membranes Ltd, 12–14 kDa molecular weight cut-off) and BNC was finally lyophilized.

### BNC characterisation

DLS experiments were performed using an ALV system (ALV-CG-3 goniometer/ALV-5000/EPP multi tau digital correlator) with a He–Ne laser (λ = 632.8 nm). The field autocorrelation functions were analyzed by the CONTIN algorithm to extract the distributions of relaxation rates^59^. The hydrodynamic radii (R_h_) distributions of BNC and NCC were obtained using the Stokes–Einstein relation.

ζ-potential measurements of BNC and NCC were conducted using a Zetasizer Nano ZS (Malvern Instruments, Worcestershire, UK) at 25 °C. ζ-potential was calculated from the measured electrophoretic mobility using the Henry equation under the Smoluchowski approximation. The results presented averages of 3 measurements made at scattering angle θ = 173°. The ζ-potential was measured in suspensions of 3.75 g/L lyophilized samples that were previously diluted with deionized water to avoid multiple scattering effects.

The BNC crystallinity was estimated by XRD analysis. Lyophilized samples were placed in capillary tubes of 0.5 mm (Hilgenberg tubes) and counted on the goniometer of a Bruker D8-VENTURE diffractometer (CuKα, λ = 1.54178 Å). The X-ray diffraction patterns were collected by performing a complete rotation (*φ* = 360°) for 180 s. The Debye–Scherrer diffraction rings, as recorded on the 2-D Photon100 detector of the instrument, were integrated to the equivalent of 2*θ* scans using the APEX3 software. The XRD graphs were created using the PROFEX graphical user interface^60^. The BNC Crystallinity index was determined based on the DIFFRAC EVA suit^61^.

### Preparation and characterization of biofilms

Aqueous dispersions of SFMPI (5% w/v) and SFMPI reinforced with either NCC or BNC (5 g nanocellulose/100 g SFMPI) were homogenized (Ultraturrax homogenizer- IKA, t 25 basic) for 2 min and placed in a hotplate stirrer (Witeg, MSH 20D) at 40 °C for 20 min. Decanted crude glycerol (43.3 g crude glycerol/100 g SFMPI) was added and the pH was adjusted to 10.5 using 5 M NaOH. The mixtures were stirred at 40 °C for 10 min and ultrasonicated for 2 min (60 kHz, 300 W, Sonoplus 3200). The final dispersions were casted into petri dishes (64 cm^2^) and dried at 30 °C for 72 h. The produced biofilms were stored at 54% relative humidity and 20 °C using a Mg(NO_3_)_2_ saturated solution for 48 h before characterization.

#### Mechanical properties

Tensile testing of the casted biofilms was conducted applying the standard method ISO 527-3:2018 Plastics—Determination of tensile properties—Part 3: Test conditions for films and sheets, International Organization for Standardization, Geneva, Switzerland^62^, in a universal testing machine Instron, Model 5900 (Instron Industrial Products, USA). Each sample was examined in sextuplicate at 23 °C and 50% relative humidity. The distance between the clamps of the grips (gauge length) was adjusted to 50 mm. The width of the biofilm strips was 10 ± 0.1 mm. Their thickness is listed in Table 1. The moving crosshead used to pull the specimens was equipped with a load cell of maximum capacity of 10 KN and the separation speed was 10 mm/min. Modulus of elasticity, tensile strength and elongation at break values were obtained by the Instron ‘Bluehill 3’ Software.

#### Water vapor permeability

WVP was determined in duplicates based on the desiccant procedure of ASTM method E 96-80^63^. Briefly, biofilms were sealed over a permeation cell with a circular opening of 0.00353 m^2^ and the system was stored in a desiccator at 23 °C. The permeation cell was filled with anhydrous silica (0% RH_pc_) while the desiccator was filled with a saturated NaCl solution (75% RH_d_) to maintain relative humidity (RH) of 75%. The weight of the system was recorded periodically over 24 h and plotted as a function of time. Slopes (g/s) were extracted via linear regression. WVTR was calculated dividing the slope by the permeation cell area. WVP (g/Pa s m) was calculated using Eq. (1):

1 $$ WVP = \frac{WVTR}{{p_{v} \times \left( {RH_{d} - RH_{pc} } \right)}} \times d $$

p_v_: vapor pressure of water at saturation at 23 °C (Pa); RH_d_: RH in desiccator; RH_pc_: RH in permeation cell; d: thickness of the film (m).

#### Solubility

Solubility was evaluated according to Zhou et al.^64^. Briefly, biofilms were dried at 105 °C for 24 h. Preweighted samples were immersed in 30 mL water and left at ambient temperature for 24 h while tubes were periodically stirred. The filtered solid fraction was oven-dried for 24 h at 105 °C until constant weight.

#### FTIR analysis

Attenuated total reflectance infrared spectroscopy (ATR-IR) measurements were performed on a Bruker Equinox 55 Fourier Transform Instrument, equipped with an attenuated total reflectance (ATR) diamond accessory, from SENS-IR and a press. The samples were placed at the center of the sample holding device and 64 scans were performed in the range 525–5000 cm^−1^ at 4 cm^−1^ resolution. Two measurements on different loaded samples were performed to confirm reproducibility.

### Fresh strawberry packaging

Freshly harvested strawberries were purchased from a local market (West Peloponnese) and used within 24 h. Off-standard in size and color berries and mechanically damaged or decayed fruits were removed. Samples were surface-sanitized by immersion in 0.5% v/v NaOCl solution^65^ for 1 min followed by rinsing with distilled water and left to drain on a filter paper. The sanitized strawberries were packed in containers sealed with SFMPI-, SFMPI-NNC- and SFMPI-BNC-based biofilms. Conventional PVC food membranes were used as control. Ten strawberries were placed into each container and various post-harvest quality parameters were determined at the beginning of the storage (Day 0) and periodically over a 15-day period of isothermal storage at 10 °C.

All methods were performed in accordance with the relevant guidelines and regulations.

#### Weight loss

The weight of each container was measured in triplicate. The weight loss of tested strawberries was determined considering the sample weight of time 0 and after x days of storage.

#### Respiration activity

The respiration activity (in terms of O_2_ and CO_2_) of the packed strawberries over 15 days of storage was recorded (days 0, 1, 3, 4, 7, 11, 15) using a headspace gas analyzer (CheckPoint 3 O_2_/CO_2_, AMETEK/MOCON Europe A/S—Denmark). For Day 0, the concentration of the gases was determined immediately after sealing.

#### Decay rate of packed strawberries

Samples were visually examined for any signs of decay e.g., color changes, microbial spoilage, softening, brown spots or wounded areas, during 15-day storage. Decay signs were recorded (days 4, 7, 15) and the decay rate was expressed as the percentage of the number of infected strawberries divided by the total number of the strawberries contained in the film-sealed container^66^.

#### Color of packed strawberries

The color was measured at five different areas of each strawberry using the colorimeter i1 Pro 3 (X-rite Pantone, United States) according to CIELAB uniform color space (days 0, 7, 15). The lightness coordinate is indicated by L^*^ parameter (black = 0, white = 100), while the a* and b* coordinates represent the green/red and yellow/blue differences respectively, with green and blue values being at the negative scale and red and yellow being at the positive scale. The cylindrical coordinates C* and h* were also recorded.

#### Firmness of strawberries

The textural quality of the stored strawberries was evaluated (days 0, 7, 15) through a puncture test using a Universal Testing Machine (Η5KS/0258, England) equipped with a 1.6 mm diameter probe. Firmness was expressed as the maximum force (N) to push the probe into a depth of 2 mm applying a load cell of 1000 N with a cross-head speed of 60 mm/min. Each berry was cut transversely into three individual parts and the test was applied in five random points at the top edge of each part. Strawberry firmness was obtained as the average of all measurements for each package sealing.

#### Chemical evaluation of strawberry quality

Chemical evaluation (days 0, 1, 3, 7, 15) was conducted on pureed and homogenized (2 min) fruits obtained using a kitchen blender. The pH of fruits was determined using a HI98100 Checker Plus pH meter (Hanna, USA). The TTA was determined according to Lan et al.^66^.

#### Microbial growth during strawberries storage

Total viable count, yeasts and molds were estimated according to Guerreiro et al.^67^. Counts were performed in triplicates after plate incubation at 25 °C for 48–72 h. Results were expressed as Log10 CFU per g of strawberry (wet basis). Microbial growth modeling was carried out using the Baranyi growth model^68^, by fitting curves using the DMFit software. Kinetic parameters including the rate (k) of microbial growth and final microbial population predicted (N_max_) were estimated at the tested packaging conditions.

### Analytical methods

Sugars, glycerol and citric acid were determined via HPLC (Prominence, Shidmadzu, Japan) equipped with a Rezex ROA-organic acid H^+^ column (300 mm length × 7.8 mm internal diameter, Phenomenex), coupled to a differential refractometer (RID-10A, Shimadzu, Kyoto, Japan). The mobile phase was a 10 mM H_2_SO_4_ aqueous solution with 0.6 mL/min flow rate at 65 °C. FAN was determined by the ninhydrin method^69^.

### Statistical analysis

Statgraphics was used for statistical analysis. The data were compared using analysis of variance (ANOVA) and Pearson’s linear correlation at 5% significance level. Significant differences between means were determined by Honest Significant Difference (HSD-Tukey test) at level of *p* < 0.05. Data were reported as mean values ± standard deviation of three independent replicates (*p* < 0.05, 95%).

## Supplementary Information

Supplementary Information.

## Data Availability

The datasets used and/or analysed during the current study are available from the corresponding author on reasonable request. The datasets are part of ongoing scientific projects and deliverables that have not yet been completed.
