# Supplementary material for: Development of biodegradable films using sunflower protein isolates and bacterial nanocellulose as innovative food packaging materials for fresh fruit preservation
Source: Sci Rep. 2022 Apr 28;12:6935. doi: 10.1038/s41598-022-10913-6 (PMC9050891; doi:10.1038/s41598-022-10913-6)
Supplement: Supplementary file 1 — Supplementary Information. [file 41598_2022_10913_MOESM1_ESM.pdf]

Figure S1

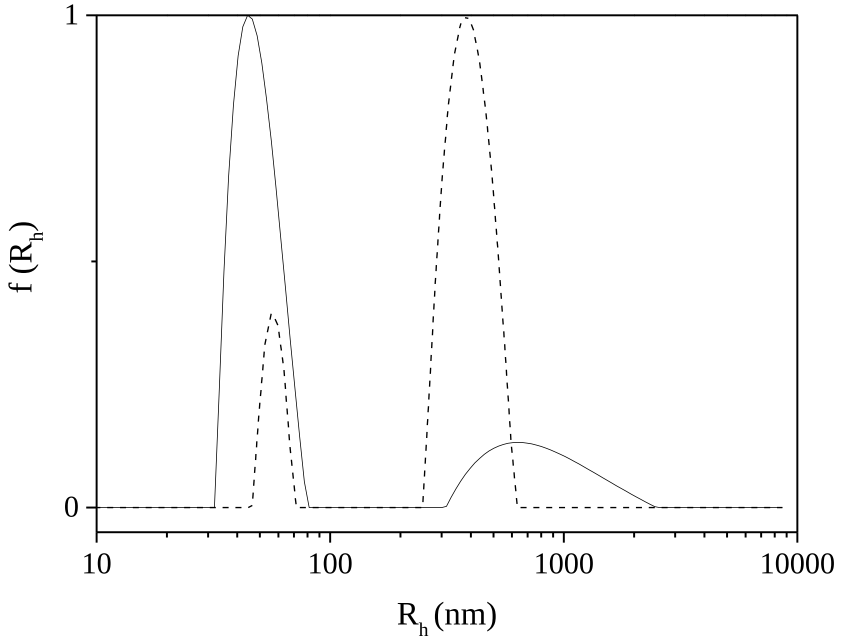

Figure S1 DLS size distribution analysis of NCC (solid line) and BNC (dash line) derived after the acid hydrolysis of BC produced by *K. sucrofermentans*

Figure S2

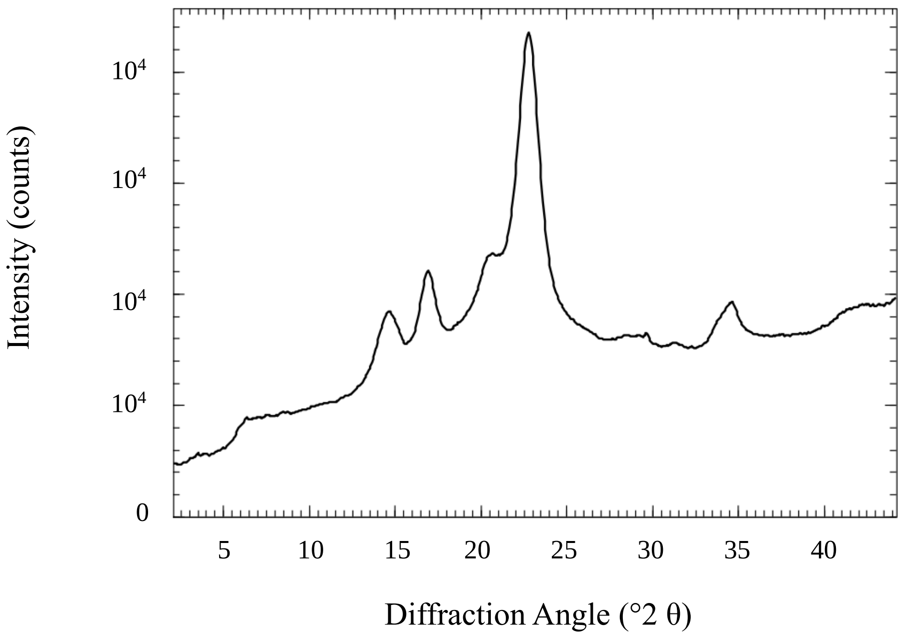

Figure S2 X-ray diffractogram of BNC derived after the acid hydrolysis of BC produced by *K. sucrofermentans*

Figure S3

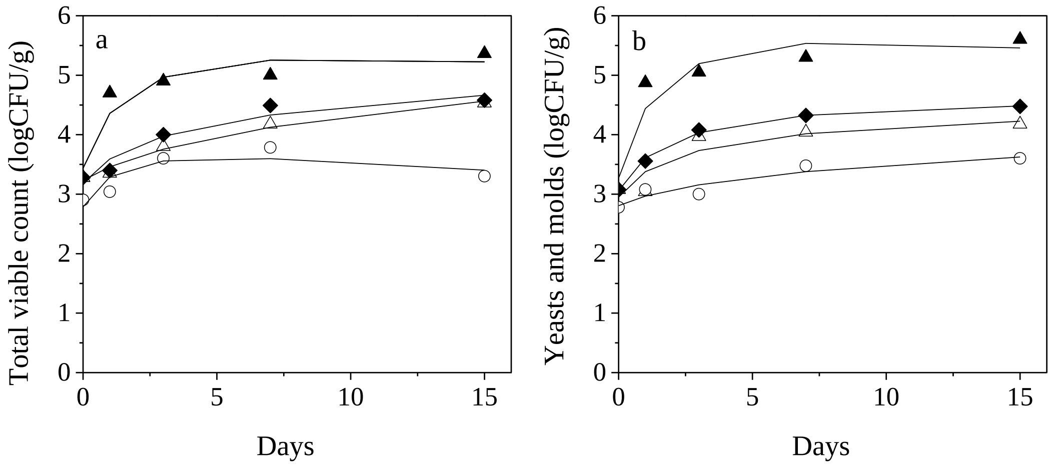

Figure S3 Microbial growth (a. total viable count, b. yeasts and molds) in strawberries sealed with PVC (▲), SFMPI- (Δ), SFMPI-NCC- (♦) and SFMPI-BNC- (○) based films over 15 days isothermal storage at 10 °C, modelled using the Baranyi growth model.

Figure S4

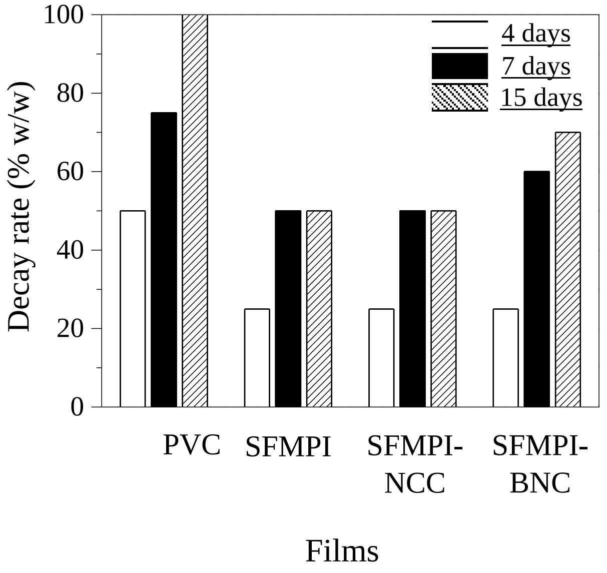

Figure S4 Decay rate of strawberry samples sealed with conventional PVC, SFMPI-, SFMPI-NCC-, and SFMPI-BNC-based films over 15 days storage at 10 °C.

Table S1 Bands of absorbance of FTIR spectra of BNC, SFMPI-, SFMPI-NCC-, and SFMPI-BNC-based biofilms.

	Absorbance band (cm^{-1})	Assignments
All samples	3000-3700	stretching of OH<br>N-H stretching vibration of hydrogen bonded amides
	2700-2995	CH stretching
	1108	C-O stretching of protein or glycosidic vibrations of ether bonds from cellulose nanocrystals
BNC	1159	asymmetric stretching vibrations from C−O−C, glycosidic vibrations of ether bonds from cellulose
	1232, 1243, 1276	out of plane bending vibration of C−O−H at C6
	1203	symmetrical stretching vibration from C−O−C
	1052	vibrations of asymmetrical C-H and stretching of C-O related to the β-glycosidic linkages between β-D-glucopyranoses in cellulose
	1643	absorbed water
	1454	H−C−H, O−C−H in-plane bending
	1425	asymmetrical angular deformation of C-H bonds
	1361	symmetric angular deformation of C-H bonding
	1031	C-O stretching at C3 position of cellulose
	919	HCH and OCH deformation from cellulose skeletal vibrations
Biofilms	1040	C-O stretching vibration of protein or C-O stretching at C3 position of cellulose
	1240	C–N stretching of amides
	1536	N-H bending of amides
	1633	C=O stretching of amide
	1741	C=O stretching of cellulosic carbonyl groups
